# Supplementary material for: Polarizable Water Model with Ab Initio Neural Network Dynamic Charges and Spontaneous Charge Transfer
Source: J Chem Theory Comput. 2025 Mar 29;21(7):3360–73. doi: 10.1021/acs.jctc.4c01448 (PMC11983713; doi:10.1021/acs.jctc.4c01448)
Supplement: Supplementary file 1 — ct4c01448_si_001.pdf [file ct4c01448_si_001.pdf]

Supporting Information

Polarizable Water Model with Ab Initio Neural Network Dynamic Charges and Spontaneous Charge Transfer

Qiujiang Liang*,†,‡ and Jun Yang*,†,‡

†*Department of Chemistry, The University of Hong Kong, Hong Kong, 999077, P.R. China*

‡*Hong Kong Quantum AI Lab Limited, Hong Kong, 999077, P.R. China*

E-mail: qliang@connect.hku.hk; juny@hku.hk

S1 Deep Neural Networks for Atomic Charges

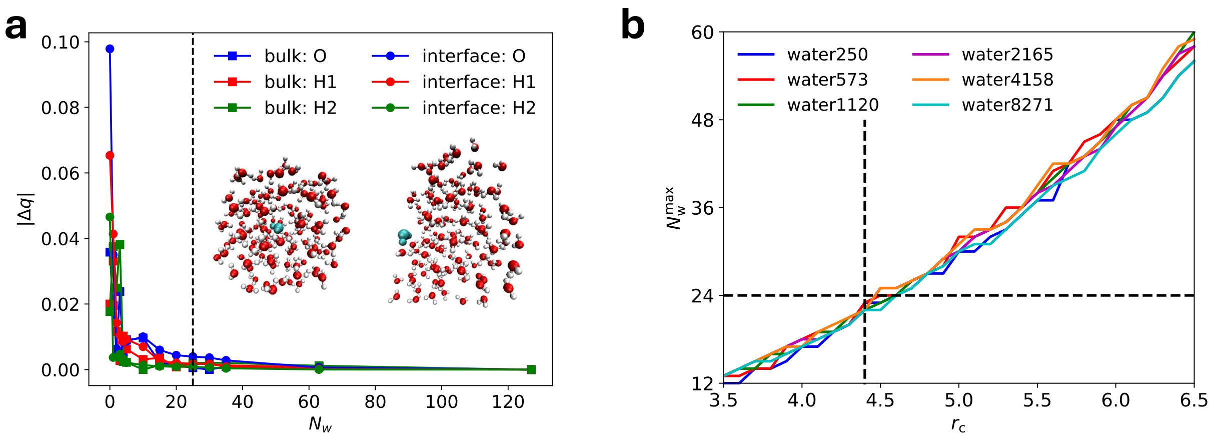

Fig. S1: (**a**) Absolute charge deviations from those with 128 surrounding water molecules as a function of number of surrounding water. The computation was conducted with MBE(3)-OSV-MP2/cc-pVTZ. (**b**) Maximal number of surrounding water molecules with various radius cutoffs.

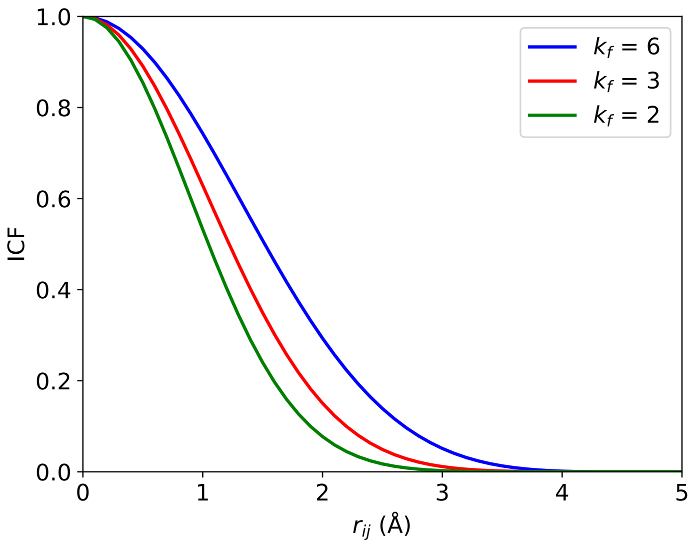

Fig. S2: The relationship between interaction classified function (ICF) and distance with various decay parameters k_f.

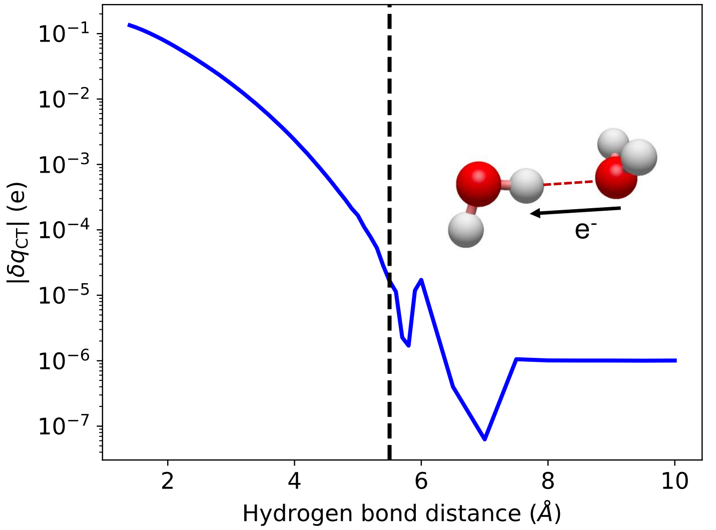

Fig. S3: The absolute transferred charges as a function of the hydrogen bond distance of a water dimer. The computation was conducted with MBE(3)-OSV-MP2/cc-pVTZ.

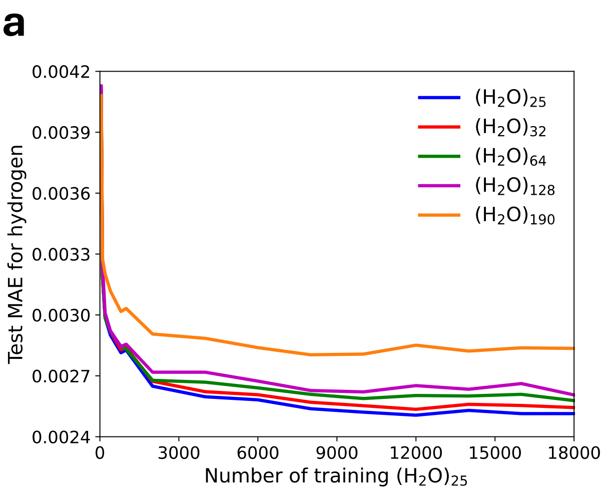

Fig. S4: Test mean absolute errors (MAEs) as a function of the number of training $(H_2O)_{25}$ for charges of (**a**) hydrogen and (**b**) oxygen.

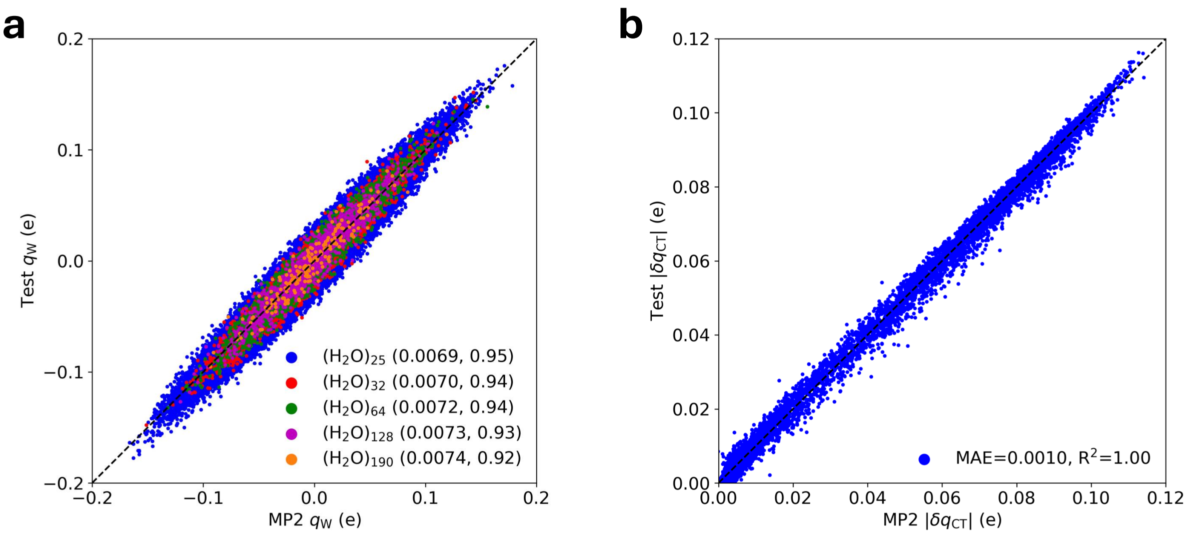

Fig. S5: Comparisons of ChargeNN charges and MP2 charges for (**a**) each water molecule as a sum of the predicted charges of the atoms within the molecule and (**b**) intermolecular charge transfer. The numbers in the right brackets are the mean absolute errors and coefficients of determinations, respectively.

S2 Water model

Table S1: Parameters of the water model

Parameters	Units	Values
k_b	$\mathrm{kj} \cdot \mathrm{mol}^{-1}\mathrm{nm}^{-2}$	366000
r^0_{OH}	nm	0.09559
k_a	$\mathrm{kj} \cdot \mathrm{mol}^{-1}\mathrm{rad}^{-2}$	372.5652
θ^0	Degree	108.3289
σ_{ij}	nm	0.3187
ε_{ij}	kj/mol	0.7331
k_c	kj/mol	35.02048
δq^0	e	0.056857

S2.1 Protocol for the benchmark tests

S2.1.1 Gas phase properties

The gas phase properties were obtained with the structures optimized by the water model.

S2.1.2 Liquid properties

To determine the liquid properties, we carried out molecular dynamics (MD) simulations with 250 water molecules within a cubic box under periodic boundary conditions. A larger water box with 2123 molecules was used to validate the reliability of the benchmark results, as demonstrated in Table S2. Langevin thermostat implemented in Atomic Simulation Environment (ASE)[1] was used to control the temperature. Monte Carlo barostat[2] was implemented for controlling the pressure. A small time step of 1 fs was adopted due to the flexible bonds and angles. A 10-Å spherical cutoff was employed for the Van der Waals and short-range Coulomb interactions. Without further specifications, the liquid properties were calculated by averaging the equilibrium values of 100 trajectories. Each trajectory's equilibrium average was taken over 300-ps snapshots following a well-equilibrated NPT run of 1 ns.

- **Enthalpy of vaporization** ΔH_{vap}: Under the assumptions that a) the volume of liquid water is negligible compared to gaseous water and water gas is ideal gas and b) the intra-molecular

potential energies are the same in both gas and liquid phases, the enthalpy of vaporization is estimated by

$$
\begin{aligned}
\Delta H_{\text{vap}} &\approx RT - (\langle U_{\text{liq}} \rangle - \langle U_{\text{gas}} \rangle) \\
&\approx RT - \left\langle U_{\text{liq}}^{\text{inter}} \right\rangle .
\end{aligned} \tag{1}
$$

In the above formula, $\langle U \rangle$ represents the average molecular potential energy.

- **Diffusion coefficient** D: The diffusion coefficient was determined by taking the slope of the mean squared displacement over time, utilizing the Einstein equation:[3]

$$
D = \frac{1}{6t} \frac{1}{N} \sum_{i=1}^{N} |r_i(t) - r_i(0)|^2 , \tag{2}
$$

where t refers to the elapsed time, N represents the number of water molecules, and $\mathbf{r}_i$ denotes the position of the center of mass of water i. The diffusion coefficient was obtained as an average over 100 20-ps NVE simulations, which were conducted using checkpoint files sampled at 1 ps intervals from 5 equilibrated NPT simulations. Notably, the diffusion coefficient was found to be dependent on the system size.[4] By analyzing the relation between the inverse of length and Diffusion coefficient, we derived the Diffusion coefficient at infinite size, using PBC boxes containing 250, 360, 512, and 2123 water molecules.

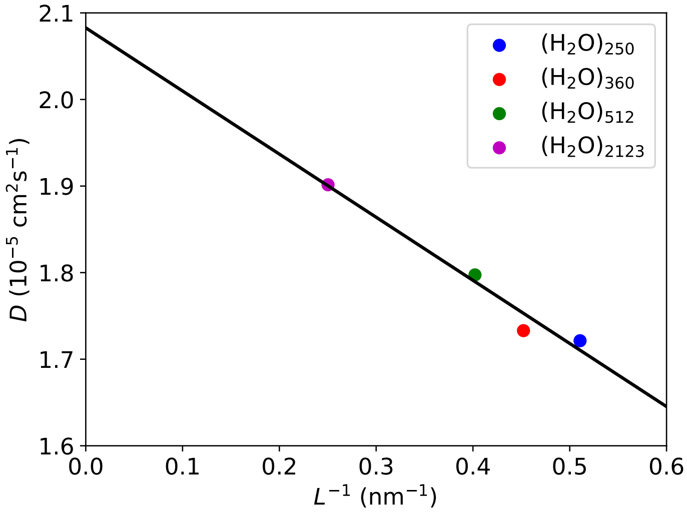

Fig. S6: Diffusion coefficients as a function of inverse box length, fitted to the inverse length of PBC boxes with 250, 360, 512, and 2123 water molecules.

- **Average molecular dipole moment** $\langle \mu_{\rm mol} \rangle$. The average molecular dipole moment was computed by setting the origin $\mathbf{r}_I^o$ to be the center of the nuclear charge of each water molecule I,

$$\langle \mu_{\rm mol} \rangle = \frac{1}{N_{\rm mol}} \sum_I^{N_{\rm mol}} \left| \sum_{i \in I} q_i (\mathbf{r}_i - \mathbf{r}_I^o) \right| . \tag{3}$$

- **Dielectric constant** ϵ. The dielectric constant was calculated with the following equation:

$$\varepsilon = 1 + \frac{4\pi}{3 \langle V \rangle k_B T} \left(\langle \mathbf{M}^2 \rangle - \langle \mathbf{M} \rangle^2 \right) , \tag{4}$$

where $\mathbf{M} = \sum_i q_i \mathbf{r}_i$ denotes the total dipole moment vector of a cell, $\langle V \rangle$ is the average volume, k_B is the Boltzmann constant and T is the simulation temperature. To get reasonable total dipole moments, the molecules were wrapped to the unit cell as their centers of mass (COMs), when the COMs were not in the PBC box. A long NPT simulation of 20 ns was carried out to get a converged dielectric constant.

- **Infrared spectrum** The infrared spectrum was obtained by performing a Fourier transform of the autocorrelation function of the total dipole moment vectors. The dipole moments were

recorded every 1 fs in one of the aforementioned 300-ps NPT trajectories.

S2.1.3 Ice melting temperature

A hexagonal (I_h) structure containing 360 water molecules was used for computing the melting point. The tetrahedrality of the ice structure was described by the average orientational tetrahedral order parameter $\langle q \rangle$ [5,6] that varies from 0 to 1,

$$\langle q \rangle = \frac{1}{N} \sum_{i}^{N} \left[1 - \frac{3}{8} \sum_{j=1}^{3} \sum_{k=j+1}^{4} \left(\cos \psi_{jik} + \frac{1}{3} \right)^2 \right], \quad (5)$$

where ψ_{jik} represents the angle between the lines connecting the oxygen i and its nearest neighboring oxygen atoms j and k. Long NPT simulations of 25 ns were carried out at 1 atm and for a range of temperatures. The melting point is identified as the $\langle q \rangle$ plunges significantly from ∼0.9 to ∼0.7, due to the phase transition.

Table S2: Comparison of liquid properties between $(H_2O)_{250}$ and $(H_2O)_{2123}$

	Units	$(H_2O)_{2123}$	$(H_2O)_{250}$	Standard deviation
N_traj		10	100	
$\langle r_\mathrm{OO} \rangle$	Angstrom	0.97	0.97	0.000
$\langle \angle_\mathrm{HOH} \rangle$	degree	106.1	106.1	0.005
ρ	g/cm^3	0.996	0.996	0.002
E_int	kcal/mol	-9.913	-9.917	0.014
H_vap	kcal/mol	10.50	10.50	0.013

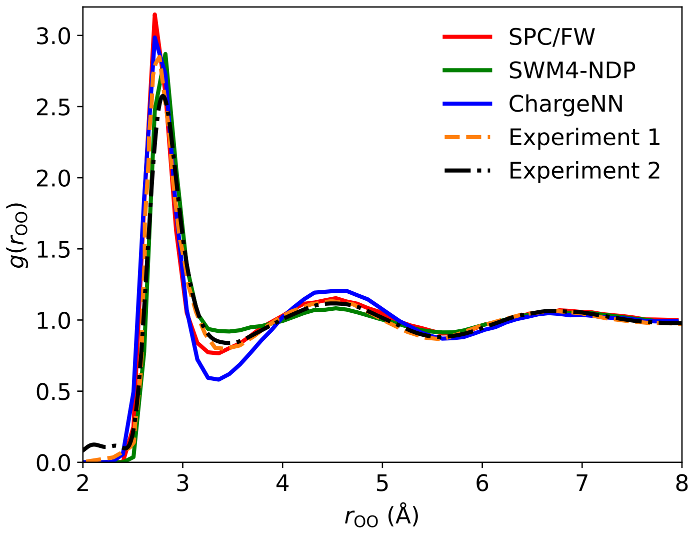

Fig. S7: Liquid radial distribution functions of the oxygen-oxygen distance obtained with SPC/FW,[7] SWM4-NDP,[8] ChargeNN and experiments[9,10] under ambient conditions.

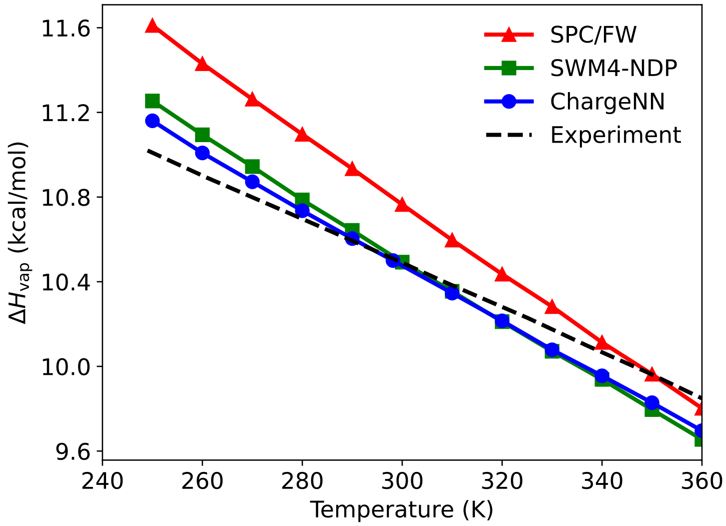

Fig. S8: Temperature dependent enthalpy of vaporization (ΔH_{vap}) computed with ChargeNN, SPC/FW, SWM4-NDP and experiment[11] at 1 atm. The ΔH_{vap} of SPC/FW were determined with $\Delta H_{\mathrm{vap}} = RT - \langle U_{\mathrm{liq}} \rangle$ as specified in ref.[7] The ΔH_{vap} of SWM4-NDP were calculated with eq. 1.

References

(1) Larsen, A. H.; Mortensen, J. J.; Blomqvist, J.; Castelli, I. E.; Christensen, R.; Dułak, M.; Friis, J.; Groves, M. N.; Hammer, B.; Hargus, C.; others The atomic simulation environment—a Python library for working with atoms. *J. Phys.: Condens. Matter* **2017**, *29*, 273002.

(2) Chow, K.-H.; Ferguson, D. M. Isothermal-isobaric molecular dynamics simulations with Monte Carlo volume sampling. *Comput. Phys. Commun.* **1995**, *91*, 283–289.

(3) Allen, M. P.; Tildesley, D. J.; others Computer simulation of liquids. *Clarendon: Oxford* **1987**,

(4) Yeh, I.-C.; Hummer, G. System-size dependence of diffusion coefficients and viscosities from molecular dynamics simulations with periodic boundary conditions. *J. Phys. Chem. B* **2004**, *108*, 15873–15879.

(5) Chau, P.-L.; Hardwick, A. A new order parameter for tetrahedral configurations. *Mol. Phys.* **1998**, *93*, 511–518.

(6) Errington, J. R.; Debenedetti, P. G. Relationship between structural order and the anomalies of liquid water. *Nature* **2001**, *409*, 318–321.

(7) Wu, Y.; Tepper, H. L.; Voth, G. A. Flexible simple point-charge water model with improved liquid-state properties. *J. Chem. Phys.* **2006**, *124*, 024503.

(8) Lamoureux, G.; Harder, E.; Vorobyov, I. V.; Roux, B.; MacKerell Jr, A. D. A polarizable model of water for molecular dynamics simulations of biomolecules. *Chem. Phys. Lett.* **2006**, *418*, 245–249.

(9) Sorenson, J. M.; Hura, G.; Glaeser, R. M.; Head-Gordon, T. What can x-ray scattering tell us about the radial distribution functions of water? *J. Chem. Phys.* **2000**, *113*, 9149–9161.

(10) Skinner, L. B.; Huang, C.; Schlesinger, D.; Pettersson, L. G.; Nilsson, A.; Benmore, C. J. Benchmark oxygen-oxygen pair-distribution function of ambient water from x-ray diffraction measurements with a wide Q-range. *J. Chem. Phys.* **2013**, *138*, 074506.

(11) Kell, G. S. Density, thermal expansivity, and compressibility of liquid water from 0. deg. to 150. deg.. Correlations and tables for atmospheric pressure and saturation reviewed and expressed on 1968 temperature scale. *J. Chem. Eng. Data* **1975**, *20*, 97–105.
